# Transcriptome and Metabolome Analyses Reveal High-Altitude Adaptation Mechanism of Epididymis Sperm Maturation in Tibetan Sheep

**DOI:** 10.3390/ani14213117

**Published:** 2024-10-29

**Authors:** Yijian Li, Yanan Yang, Binyan Yu, Rong Gao, Xinrong Wang

**Affiliations:** College of Animal Science and Technology, Gansu Agricultural University, Lanzhou 730070, China; liyijian2023@126.com (Y.L.); yangyn@gsau.edu.cn (Y.Y.); 17899317230@163.com (R.G.)

**Keywords:** epididymis, high altitude, sperm, *SYCP2*, cAMP signaling pathway

## Abstract

In this study, the epididymal histology, caepididymal sperm physiological parameters, serum reproductive hormones, and antioxidant enzyme SOD levels of Tibetan sheep at a 2500 m and 3500 m altitude were compared by using a combination of transcriptome and metabolomics methods. This was conducted to investigate the effects of a high-altitude environment on spermatogenesis and the maturation of Tibetan sheep. The results showed that compared to the low-altitude group, the high-altitude group had a smaller epididymal lumen, thicker epididymal wall, significantly decreased sperm survival rate, and significantly increased sperm deformation rate, but no difference in sperm motility and sperm respiratory intensity. With increasing altitude, Tibetan sheep showed a decreasing trend in serum reproductive hormones (FSH and T), while the antioxidant enzyme SOD activity was significantly reduced. Transcriptomic analysis revealed 139 differentially expressed genes in the Tibetan sheep epididymis under high-altitude conditions. The *SYCP2* gene is involved in multiple biological processes related to reproduction and plays an important role in the regulation of epididymal function and sperm quality in Tibetan sheep. Genes like *ADCYAP1R1*, *CABP2*, *CALN1*, and *ATP6V1B1* can help maintain sperm viability and maturation by regulating the cAMP signaling pathway, calcium ion homeostasis, and cellular signaling. Metabolomic analysis found that the high-altitude group had increased adenosine content and decreased prostaglandin I2 content in the epididymis. These metabolites are involved in spermatogenesis, motility, fertilization, and early embryonic development. The integrated omics analysis suggests that Tibetan sheep adapt to the high-altitude hypoxic environment by regulating cAMP signaling pathway genes like *ADCY* and *PRKACA*, as well as metabolites like adenosine and prostaglandin I2, to maintain epididymal function and sperm motility. These genes and metabolites play an important role in maintaining normal epididymal function and sperm motility at high altitudes.

## Simple Summary

This study investigated the effects of a high-altitude environment on the epididymis and sperm maturation of Tibetan sheep. Transcriptomic and metabolomic analyses showed that the epididymal tail of Tibetan sheep reared at different altitudes exhibited differential gene expression and metabolite profiles related to key signaling pathways involved in reproduction, immunity, and sperm motility and maturation. The high-altitude environment led to decreased superoxide dismutase (SOD) activity, reduced testicular and epididymal weights, as well as decreased sperm quality and quantity in Tibetan sheep. However, Tibetan sheep maintain sperm motility by regulating metabolic pathways such as glycolysis and ATP production, thereby adapting to the high-altitude hypoxic environment. These adaptive responses in the epididymis are an important physiological mechanism that allows Tibetan sheep to survive in the high-altitude environment.

## 1. Introduction

The low reproductive ability of Tibetan sheep in high-altitude areas is an important factor limiting the development of the local animal husbandry industry [1]. The extreme weather and harsh environment in this region have a limiting effect on the reproductive performance of local sheep to a large extent, which is especially obvious for male rams. This limiting effect is mainly reflected in the aspects of the testicular seminiferous tubules and spermatogenesis [2,3]. Spermatozoa are produced from the seminiferous epithelium in the testes, and after development and metamorphosis, spermatozoa migrate to the epididymis, where they enter the next stage of maturation and are stored [4,5,6]. The endothelium of the epididymal wall consists of multiple types of cells, such as principal, basal, narrow, lineage, and halo cells, which play an important role in regulating sperm maturation prior to fertilization [7,8]. Sperm maturation in the epididymal lumen involves multiple biochemical reactions, most of which are currently unknown. Therefore, it is important to investigate the development and maturation process of spermatozoa in the epididymis of sheep in high-altitude regions for the development of the sheep industry in alpine regions.

Transcriptome sequencing has become the most widely adopted method for correlating biological phenotypes with underlying molecular mechanisms. The detailed application of transcriptomics in the field of animal reproduction provides deep molecular insights into the key stages and organizations of the reproductive system. Relevant studies have utilized RNA-seq technology to screen *EPHB6*, *TLR1*, *MUC20*, etc., which are key genes that may regulate the functions related to epididymal cell homeostasis, innate immunity, differentiation, motility, and transporter and sperm maturation, and whose differential expression may affect the reproductive performance of the epididymis [9]. Meanwhile, differentially expressed genes (DEGs) in the head of the epididymis and sperm maturation in yak and cattle were investigated, and *DEFB109*, *DEFB121*, *DEFB123*, *DEFA1*, *LY6G5C*, *SLC13A2*, *CST3*, *CRYBA4*, and *ADM28* were found to be associated with innate immune response, spermatid maturation, motility, and antimicrobial function [10]. Metabolites provide regulatory control of gene transcription and protein expression as the end result of gene transcription and protein expression [11]. Alterations in low-molecular-weight metabolites can serve as indicators of bioturbation caused by disorders in metabolic pathways [12]. Many researchers have extensively studied metabolite information in the epididymis and semen through metabolomics [13,14,15]. These studies have elucidated the involvement of fatty acids in sperm membrane structural organization, energy metabolism, and signaling molecules, and have demonstrated that spermatozoa can derive energy from fatty acid oxidation. In addition, it has been shown that amino acids and polyamines may have an effect on spermatogenesis and maturation [16,17]. Several studies have examined the correlation between seminal plasma metabolites and sperm quality, revealing that certain metabolites, such as leucine, taurine, and carnitine, are associated with the fluid storage capacity of sperm [18,19]. The development of the epididymis and its physiologic functions are influenced by the environment. However, few studies have investigated the genes and metabolites regulating the epididymis in Tibetan sheep at different altitudes. The combined analysis of the transcriptome and metabolome can help to understand the regulatory mechanism at the molecular level and provide a basis for improving animal reproductive performance. Therefore, it is necessary to combine multi-omics data to understand the altitudinal differences in the transcriptional metabolism of epididymis in sheep and explore the reproductive potential of Tibetan sheep.

High-altitude Tibetan sheep (more than 3800 m above sea level, TSH) and low-altitude Tibetan sheep (2500 m above sea level, TSL) were selected for this study. Histological and morphological observations of the epididymis of the Tibetan sheep were carried out to analyze the DEGs and differentially expressed metabolites (DEMs) related to sperm survival rate and sperm respiration in different altitudes through the joint analysis of the transcriptome and metabolome, analyzing the mechanism of the role of the epididymis in the reproductive process of Tibetan sheep. This study contributes to improving the reproductive ability of Tibetan sheep and increasing the population of Tibetan sheep with a high reproductive ability.

## 2. Materials and Methods

### 2.1. Statement of Ethics

Experiments involving Tibetan sheep were carried out in accordance with the Chinese legislation on the use and care of laboratory animals. All experimental procedures involving animals were approved by the Animal Care Committee of Gansu Agricultural University.

### 2.2. Animal Breeding and Sample Collection

In this experiment, Tibetan ram sheep were randomly selected from Hualong County (low-altitude group, TSL) and Haiyan County (high-altitude group, TSH) in Qinghai Province. All lambs were immunized according to standardized procedures before weaning at 56 days of age. They were kept in individual pens of 0.8 × 1 m, and all test individuals were subjected to the same feeding conditions until 12 months of age. Six Tibetan sheep were randomly selected from each of the two groups for slaughter and subsequent trials. After slaughtering, we collected the left epididymis. The weight and morphology of the long and short axes of the individuals’ epididymis were measured using a soft tape measure (accuracy of 1 mm), vernier calipers (0–150 mm range), and electronic scales (accuracy of 0.01 g).

### 2.3. Semen Quality Identification and Hematoxylin–Eosin Staining of Epididymal Tissue

Epididymal sperm were collected by cutting and incubated for 15 min with in vitro fertilization reagents. Semen parameters, including sperm survival rate (%), sperm motility (%), sperm respiratory intensity (sec), and sperm deformation rate (%) were assessed. Sperm survival rate was determined by eosin black staining. Sperm motility was determined by observing the motility performance under a microscope. Sperm respiratory intensity was determined using the fading time of methylene blue reagent (taking 1 drop of methylene blue solution and 1 drop of semen on a slide). After mixing, the mixture was inhaled into two sections of capillary tube about 2~3 cm, one section was placed at 37~40 °C, and the time needed to observe the fading of methylene blue was compared. The sperm deformation rate was observed by making sperm sheets and using H&E staining. Paraffin sections containing epididymal tissue were obtained. The sections were then degreased and hydrated to remove the wax and return them to a hydrated state. Next, pre-staining was performed, including dehydration and degreasing steps. Subsequently, the treated sections were stained sequentially with hematoxylin and eosin staining solutions. After staining was complete, the sections were dehydrated and fixed on slides with gradually changing ethanol concentrations. Finally, the slides were covered with seals and observed with a microscope.

### 2.4. Serum Hormone Determination

The sheep hormone levels in the specimens were determined using the double antibody sandwich method, which includes follicle-stimulating hormone (FSH), testosterone (T), and superoxide dismutase (SOD). A purified sheep hormone capture antibody was coated with a microtiter plate to make a solid-phase antibody. Sheep serum was added to the coated microtiter wells sequentially, and then combined with HRP-labeled detection antibody to form an antibody–antigen–enzyme-labeled antibody complex. The complex was washed thoroughly and then washed with the substrate TMB to develop the color. TMB changes to blue under the effect of the HRP enzyme, and then changes to a final yellow color under the effect of acid. The shade of color was positively correlated with the sheep hormone in the sample. The absorbance (OD value) was measured at 450 nm with an enzyme marker, and the contents of sheep hormone in the sample were calculated using a standard curve.

### 2.5. RNA-Seq and Bioinformatics Analysis

RNA-seq and bioinformatics analysis were performed using the Trizol kit (Invitrogen Life Technologies, Shanghai, China) to extract the total RNA from the epididymal samples from Tibetan sheep, and a NanoDrop spectrophotometer (Thermo Scientific, Waltham, MA, USA) was used to assess the concentration, quality, and integrity. The enrichment of polyA tail mRNA was achieved using Oligo(dT) magnetic beads, followed by random interruption using divalent cations. The fragmented mRNA served as a template for cDNA synthesis using random oligonucleotide primers. The double-stranded cDNA was purified and dichotomously repaired to introduce the “A” base at the 3′ end, and sequencing junctions were attached. Subsequently, the cDNA libraries were screened using AMPure XP microspheres targeting 400–500 bp sized PCR fragments and further purified using AMPure XP microspheres to obtain the final libraries. Differential expression analysis was performed on the comparative combinations using DESeq (v1.38.3) software. Differential analysis of the gene lists was performed using DESeq, and the selection criteria for identifying differentially expressed genes (DEGs) were absolute value fold change > 1 (|log2FoldChange| > 1) and a significant *p*-value of less than 0.05. Functions of DEGs were determined using the Gene Ontology (GO) and Kyoto Encyclopedia of Genes and Genomes (KEGG) databases (http://geneontology.org/, accessed on 30 May 2024, http://www.kegg.jp, accessed on 30 May 2024). Significantly enriched GO terms and KEGG pathways were identified based on a statistical significance threshold of *p* < 0.05.

### 2.6. GC-LC/MS Analysis

Immediately after dissection, 80 mg of cauda epididymis tissue was cut on dry ice and loaded into Eppendorf tubes (2 mL). The tissue was homogenized using a homogenizer with 200 μL H_2_O and 5 ceramic beads to homogenize the tissue samples. Then, 800 μL of methanol/acetonitrile (1:1, *v*/*v*) was added to the homogenate for metabolite extraction. The mixture was centrifuged for 20 min (14,000× *g*, 4 °C). The supernatant was dried in a vacuum centrifuge. For LC-MS analysis, the sample was redissolved in 100 μL of acetonitrile/water (1:1, *v*/*v*) solvent, centrifuged at 14,000× *g*, at 4 °C for 15 min, and the supernatant was saved. The supernatant was then injected into an ultra-high performance liquid chromatography (UHPLC) system (1290 Infinity LC, Agilent Technologies, Santa Clara, CA, USA) coupled with a quadrupole time-of-flight mass spectrometer (AB Sciex TripleTOF 6600, AB Sciex, Shanghai, China). This analytical platform was used to perform composition and mass spectrometry (MS) analysis of the metabolites in the caudal part of the epididymis collected from Tibetan sheep populations living at different altitudes. The analysis was conducted at Shanghai Personal Biotechnology Co (Shanghai, China). A 2.1 mm × 100 mm ACQUIY UPLC BEH Amide 1.7 μm column (Waters, Wexford, Ireland) was used for HILIC separation. In the ESI positive and negative modes, the kinetic phase consisted of A = an aqueous solution of 25 mM ammonium acetate and 25 mM ammonium hydroxide, and B = acetonitrile. The gradient started at 95% B for 0.5 min, then decreased linearly to 65% in 6.5 min, and then to 40% in 1 min for another minute. This was then increased to 95% in just 0.1 min, followed by a re-equilibration period of 3 min. The ESI source conditions were set as follows: ion source gas 1 (Gas1) and ion source gas 2 (Gas2) were both set to 60, the curtain gas (CUR) was set so that the source temperature was maintained at 600 °C, and the ion voltage float (ISVF) was ±5500 V. In MS-only acquisitions, the instruments was set to acquire over the m/z range 60-1000 Da, and the accumulation time for TOF MS scan was set at 0.20 s/spectra. The parameters were fixed as follows: the collision energy (CE) was a constant value of 35 V, ±15 eV; the declustering potential (DP) values were +60 V (+) and −60 V (−); isotopes were excluded; 10 candidate ions were monitored per cycle. 

After summation and normalization, the processed data were analyzed using R packages (ropls). Subsequently, multivariate data analysis techniques were applied, including Pareto-scale principal component analysis (PCA) and orthogonal partial least squares discriminant analysis (OPLS-DA). The robustness of the model was assessed using the 7-fold cross-validation and response alignment tests. The contributions of each variable to categorization were determined by calculating the value of the projected importance (VIP) of each variable in the OPLS-DA model. The significance of differences between two independent groups of samples was determined using Student’s *t*-tests. Metabolites showing significant changes were screened using VIP > 1 and a *p*-value < 0.05. Pearson’s correlation analysis was performed to assess the relationship between the two variables.

### 2.7. Combined Metabolomic and Transcriptomic Analysis

We first obtained the results of the quantitative detection and analysis of metabolomics and transcriptomic data. Subsequently, correlation and O2PLS analyses were performed. After extracting the metabolites and differentially expressed transcripts, the relevant enzymes corresponding to the metabolites in the KEGG database (https://www.kegg.jp, accessed on 30 May 2024) were identified based on the results of the correlation analysis. Differential enrichment analysis was used to identify the corresponding differentially expressed metabolites between the two histological datasets and to finalize the shared annotation pathways of the differential metabolites and genes. Gene and metabolite associations in common metabolic pathways were visualized using Cytoscape (V3.3.0) software. Pearson correlation analysis was used to investigate the correlation of some DEGs and DMs with phenotypic traits.

### 2.8. RT-qPCR Validation

The RNA-seq results were validated by selecting 9 DEGs (*NOTUM*, *SLC18A2*, *PI3*, *RNASE*, *POU5F1*, *CSMD1*, *MYBPH*, *GRIA1*, and *DPYS*) for qRT-PCR. The internal reference gene *GAPDH* was used for normalization. Primers for qRT-PCR were designed using Primer software (version 5.0) (Table S1) and analyzed using qRT-PCR in the Roche LightCycler96 BR Green Pro Taq HSPCR Kit (Changsha, Hunan, China). Relative expression levels were calculated using the 2^−ΔΔCt^ method. Statistical analyses were performed using SPSS 22.0 software (SPSS., Chicago, IL, USA) with one-way analysis of variance (ANOVA). Statistical significance was determined as *p* < 0.05. 

### 2.9. Data Statistics and Analysis

We utilized SPSS version 26.0 (IBM Corp., Armonk, NY, USA) to conduct independent variance *t*-tests and correlation analyses. The data are reported as the means ± standard deviations. For O2PLS analysis and histogram creation, we employed Omic-Share Tools (https://www.omicshare.com/, accessed on 30 June 2024) and the Chiplot website (https://www.chiplot.online/, accessed on 30 June 2024).

## 3. Results

### 3.1. Histological Observations of Epididymis and Semen Quality

**Figure 1:**
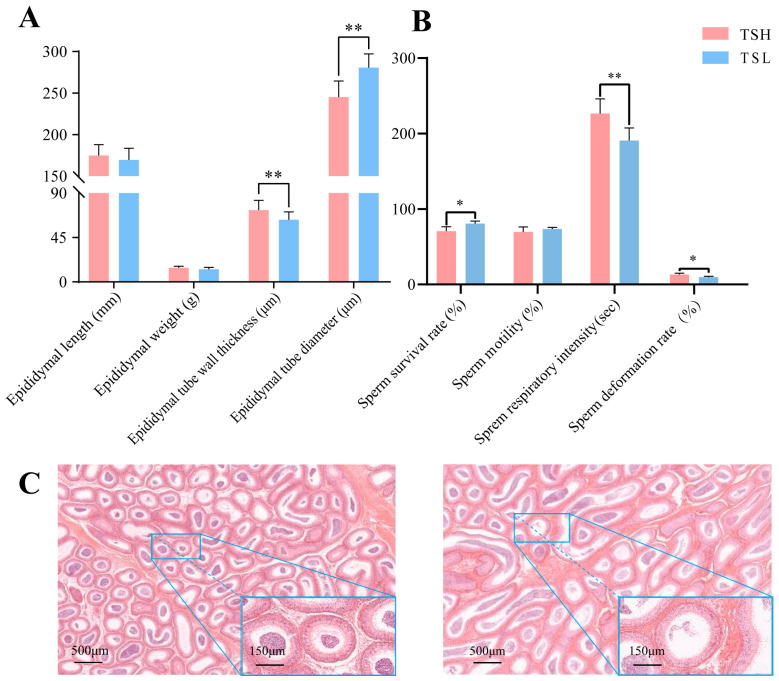
(**A**) Morphological data of epididymis in Tibetan sheep at different altitudes. (**B**) Identification of sperm quality in epididymis of Tibetan sheep at different altitudes. TSH: high-altitude Tibetan sheep; TSL: low-altitude Tibetan sheep. (**C**) HE staining of the caudal part of the epididymis in Tibetan sheep at different altitudes. Left: high-altitude Tibetan sheep flock. Right: low-altitude Tibetan sheep flock. * *p* < 0.05, ** *p* < 0.01.

The results of the epididymal histomorphometry show that there were significant differences in the epididymal morphology between the Tibetan sheep at different altitudes (*p* < 0.05). In the TSH group, the epididymal duct wall thickness was significantly higher than that of the TSL group (*p* < 0.05), but the epididymal duct diameter was significantly lower than that of the TSL group (*p* < 0.05) (Figure 1A). Measurements of the epididymal length and weight showed no significant differences between these two groups. Meanwhile, the results of the sperm quality characterization indicate that the sperm survival rate of the TSH group was significantly lower than that of the TSL group (*p* < 0.05), while the sperm respiratory intensity was highly significantly lower than that of the TSL group (*p* < 0.01, Figure 1B). The results of HE staining of the epididymal tissues demonstrate that the caudal lumen area of the epididymis of the TSH Tibetan sheep was relatively small and the number of lumens was dense, while the epididymal duct wall in the TSH group was thicker than that in the TSL group (*p* < 0.01, Figure 1C). The above results indicate that the TSH reproductive performance was weaker than that of the TSL, which might be related to their epididymal morphology and sperm quality.

### 3.2. Serum Hormone Levels

The serum hormone contents of Tibetan sheep at different altitudes were compared (Table 1). The differences in the serum contents of FSH and T were not significant between the high- and low-altitude Tibetan sheep, but the SOD content of the TSL group was significantly higher than that of the TSH group (*p* < 0.01).

### 3.3. Identification of DEGs

**Figure 2:**
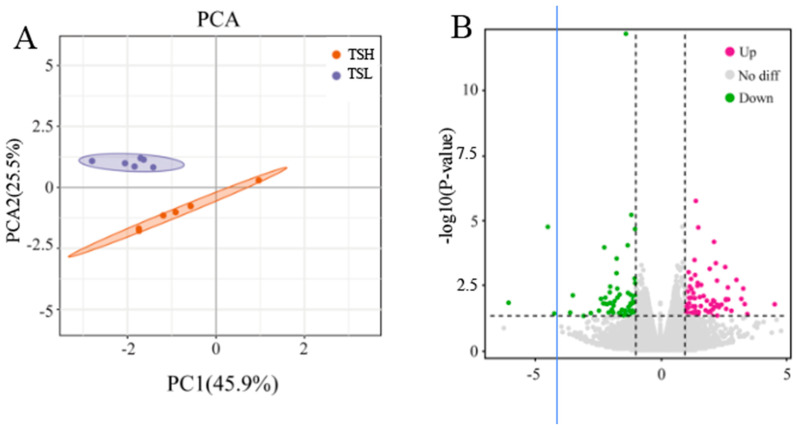
(**A**) Principal component analysis. (**B**) Volcano maps of DEGs in both TSL and TSH.

**Figure 3:**
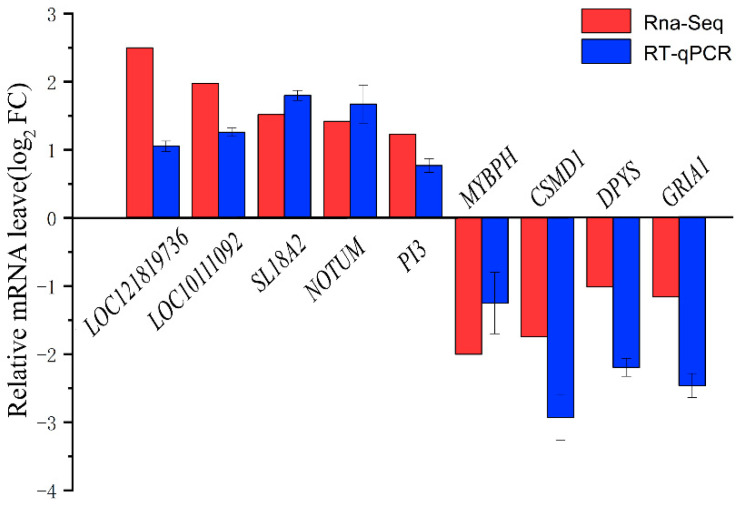
Validation of DEGs using RT-qPCR.

The clean data for each sample amounted to approximately a Q30 base percentage over 94.28%. Twelve independent cDNAs were constructed using epididymal tissue RNA. The clean read length localization rate for the sheep reference genome was 93.00–95.00%. Approximately 90% of the read fragments were uniquely mapped and used for subsequent analysis (Table S2). A total of 139 DEGs were identified in the TSL and TSH groups, respectively (Figure 2B). The qRT-PCR results were validated to confirm the trends observed in the RNA-seq data (Figure 3).

### 3.4. Functional Enrichment of DEGs

**Figure 4:**
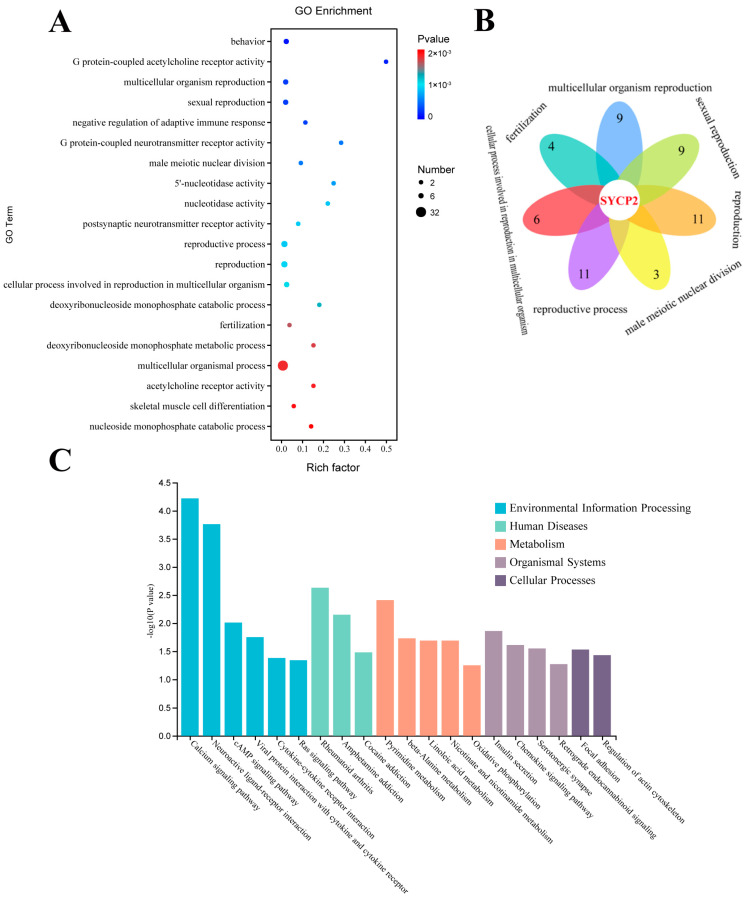
(**A**) Factor map of GO-enriched entries. (**B**) DEGs in all seven GO entries related to reproduction. (**C**) KEGG pathway significantly enriched for DEGs.

Gene ontology (GO) analysis was used to investigate the role of DEGs in the epididymal tissues of Tibetan sheep at different altitudes. The analysis focused on the biological processes, with the categories described in the results (Figure 4A). GO enrichment analyses revealed a higher number of functional terms related to biological functions compared to cellular components and molecular functions. The top five terms in each category with the highest number of DEGs included biological processes (multicellular biological processes, multibiotic processes, multibiotic reproductive processes, reproduction, and reproductive processes), cellular components (membrane components, membranes, membrane parts, extracellular regions, and components of the plasma membrane), and molecular function (transmembrane signaling receptor activity, signaling receptor activity). Meanwhile, we found that *SYCP2* was differentially expressed in TOP20 GO entries (Figure 4B). KEGG enrichment analysis showed that these genes were significantly enriched in the neuroactive ligand–receptor interaction pathway, calcium signaling pathway, cAMP signaling pathway, and cytokine–cytokine receptor interactions (Figure 4C).

### 3.5. Differential Metabolites

**Figure 5:**
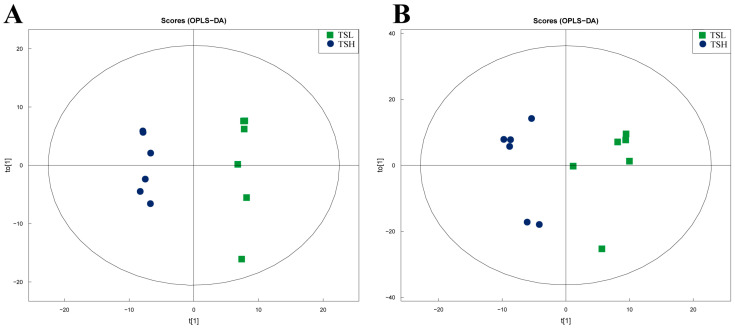
Plot of OPLS-DA model scores for both groups of samples. (**A**) NEG; (**B**) POS.

**Figure 6:**
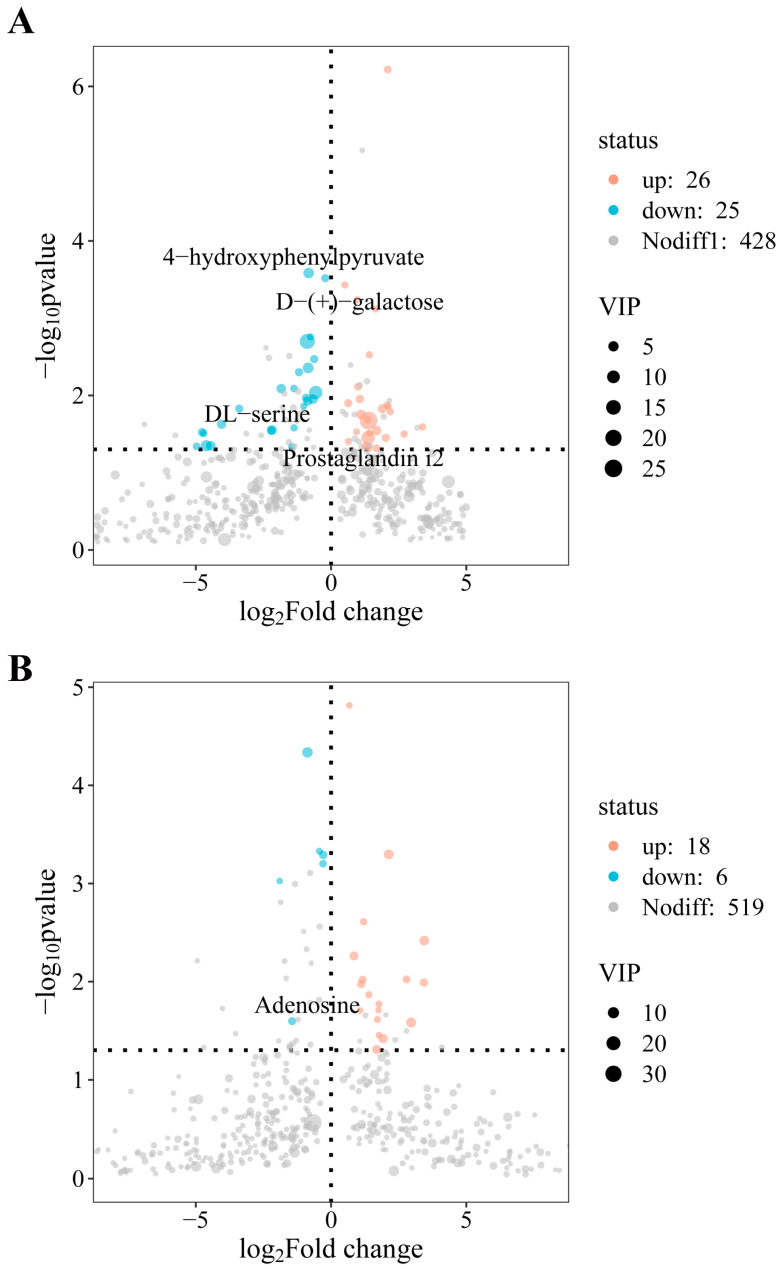
(**A**) Volcano plots of differential metabolites in positive ions in TSL vs. TSH. (**B**) Volcano plots of differential metabolites in negative ions in TSL vs. TSH.

OPLS-DA analysis showed significant separation between the sample groups, and the metabolites in the epididymis of sheep at different altitudes fell within the 95% confidence interval (Figure 5A,B). The developed model demonstrated stability and reliability and was able to effectively compare the differences between the two groups. By applying VIP > 1 and q < 0.05 as the screening criteria (DM), we identified 36 positive-ion DMs in the TSL and TSH, respectively, as well as 75 negative-ion differential metabolites in these groups (Figure 6A,B). Notably, the levels of D-(+)-actose, Pro2, and 1-(3,4-dichlorophenyl)-3,3-dim were significantly higher in the TSL group compared to the TSH group; conversely, dantrolene, xylitol, and 4-hydroxyphenylpyranilate were significantly lower (*p* < 0.05).

### 3.6. Functional Enrichment of Differential Metabolites

**Figure 7:**
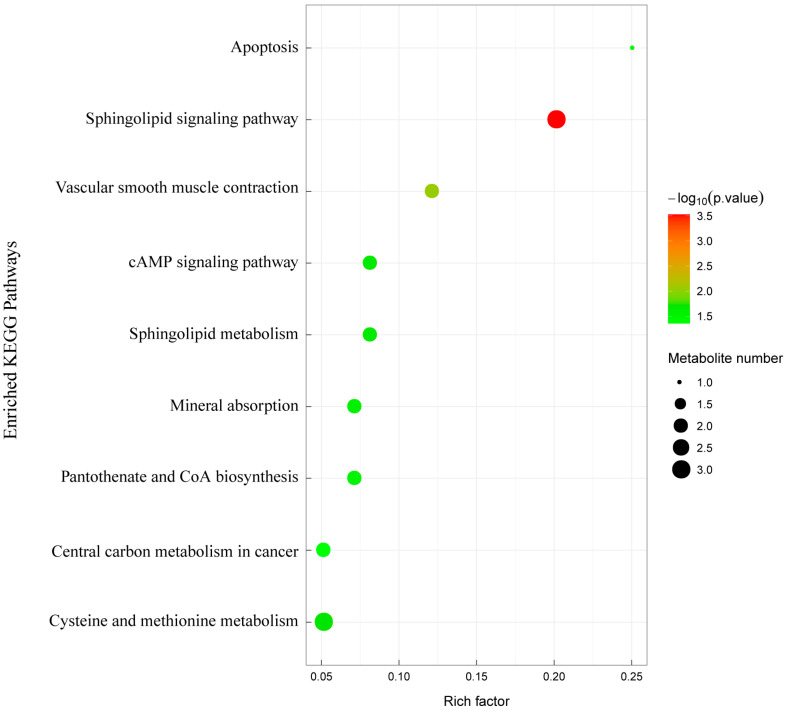
KEGG pathway was significantly enriched for differential metabolites.

KEGG pathway analyses revealed differences mainly in the metabolic pathways (cysteine and methionine metabolism, sphingolipid metabolism, and pantothenate and coenzyme A biosynthesis), environmental information processing (sphingolipid signaling pathway, cAMP signaling pathway, and VEGF signaling pathway), and alterations in biological systems (vascular smooth muscle contraction and mineral uptake) (Figure 7).

### 3.7. Integrated Transcriptome and Metabolome Analysis

**Figure 8:**
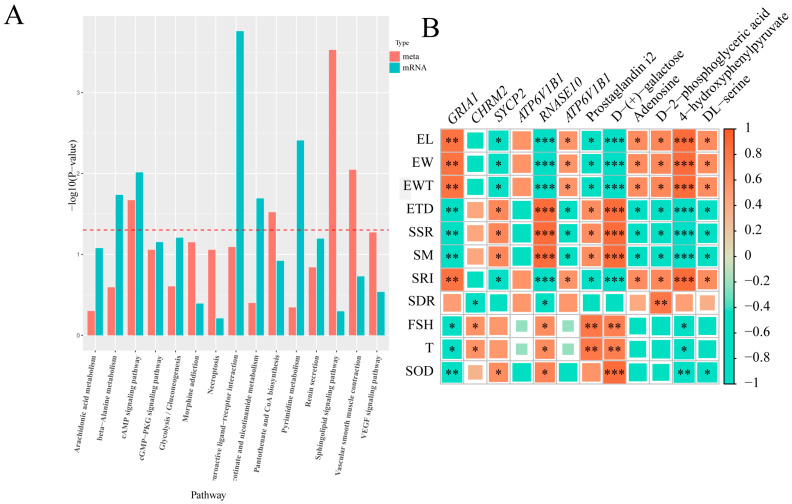
(**A**) Metabolomic and transcriptomic differences in co-enrichment pathways. The results above the red dashed line are those with *p* < for 0.05. (**B**) A correlation analysis of some DEGs and DMs with phenotypic traits. * *p* < 0.05, ** *p* < 0.01, and *** *p* < 0.001. EL: epididymal length; EW: epididymal weight; EWT: epididymal tube wall thickness; ETD: epididymal tube diameter; SSR: sperm survival rate; SM: sperm motility; SRI: sperm respiratory intensity; SDR: sperm deformation rate.

The analysis of the shared pathways between DMs and DEGs showed (Figure 8A) that in the cAMP signaling pathway and VEGF signaling pathway, prostaglandin I2 was significantly increased in the TSM group compared to the TSH group, while the expressions of the GRIA1 and CDC42 genes were significantly down-regulated. In the glycolysis/gluconeogenesis pathway, the D-2-phosphoglyceric acid level was significantly decreased, and the expressions of the PGK and PDHA genes were significantly up-regulated in the TSL group compared to the TSH group (Figure 8B). Pearson correlation analysis revealed significant positive correlations between the GRIA1 and ATP6VIB1 genes with D-2-phosphoglyceric acid, 4-hydroxyphenylpyruvate, and DL-serine, as well as with the epididymal length, epididymal weight, epididymal duct wall thickness, and sperm respiratory intensity (*p* < 0.05). In addition, D-2-phosphoglyceric acid was positively correlated with the sperm deformation rate (*p* < 0.05). Additionally, ASZ1, CDC42, PDHA2, and PHGDH showed significantly positive correlations with prostaglandin I2, sperm survival rate, sperm motility, and epididymal duct diameter (*p* < 0.05).

## 4. Discussion

High altitudes may affect the development of animal organs. It has been shown that lung volume is larger at high altitudes than at low altitudes [20], and that the heart-to-body weight ratio of Tibetan sheep increases with increasing altitude [21]. In addition, in a histological comparison of organs in animals at different altitudes, it was found that the pulmonary artery volume, wall thickness, and elastic fiber content increased consistently with altitude [21]. In studies on the renal aspect, the kidneys of rats living at high altitudes were found to exhibit glomerular congestion, interstitial congestion, glomerular necrosis, tubular atrophy, tubular vacuolization, tubular necrosis, tubular lumen cast formation, and inflammatory cell infiltration [22]. Reproductive ability plays a key role in livestock production, but the low-temperature hypoxic environment at high altitudes will have an impact on the development of animal reproductive organs, and high-altitude environments will inhibit follicular development and ovulation in Tibetan sheep [23]. In males, high altitude causes decreased testicular and epididymal weights, abnormal levels of reproductive hormones, excessive germ cell apoptosis, and decreased sperm concentration [24]. The caudal part of the epididymis is the key to sperm maturation and storage. In this experiment, we found that the sperm survival rate and respiratory intensity of Tibetan sheep in the TSH group were significantly lower than those in the TSL group, which is consistent with the findings of Verratti et al. [25]. At the same time, the rate of sperm malformation increased significantly with the increase in altitude. However, the molecular effect of a plateau environment on epididymal development and spermatogenesis in Tibetan sheep is not clear. In the present study, comparative transcriptomic and metabolomic analyses of epididymal tissues from Tibetan sheep exposed to high and low altitudes were performed using RNA-seq technology.

The epididymal morphology and sperm quality showed significant differences between Tibetan sheep at high and low altitudes. In the high-altitude group (TSH), the epididymal duct wall thickness was significantly higher, while the epididymal duct diameter was significantly lower compared to the low-altitude group (TSL). However, no significant differences were observed in epididymal length and weight between the two groups. The decline in reproductive performance of Tibetan sheep at high altitudes is likely due to the combined effects of the hypoxic environment and temperature fluctuations. Studies have shown that the low oxygen content at high elevations may impair sperm development and maturation, while the temperature variations could disrupt normal epididymal function [26]. As a physiological adaptation mechanism, Tibetan sheep may regulate the morphology of the epididymis and sperm quality to cope with these environmental stressors [27,28]. However, this adaptive response may come at the cost of reduced fertility. In this study, a total of 139 genes showed differential expression patterns. Functional bioinformatics analysis based on GO and KEGG databases revealed that these different genes are mainly involved in processes and pathways related to multicellular organism processes, reproduction, immunity, and membrane components. Notably, differentially expressed genes were significantly enriched in key signaling pathways, such as the calcium signaling pathway, cAMP signaling pathway, and cytokine–cytokine receptor interactions. Interestingly, our results show that environmental information-processing genes were most abundant in the epididymis tails of Tibetan sheep at different altitudes. In addition, we identified several up-regulated genes in the TSM group that are epididymally involved in functions related to sexual reproduction, including *SYCP2* and *RNASE10*. The protein encoded by the *SYCP2* gene is involved in homologous chromosome pairing and genetic recombination during meiosis in germ cells [29]. It is a key component of homologous chromosome binding and helps to ensure that chromosomes are correctly paired and genetically recombined to produce diverse genetic information. Therefore, the expression level and function of the *SYCP2* gene are critical for normal meiosis and spermatogenesis. Consistent with the results of this experimental study [30], another study found that a decrease in *SYCP2* may lead to a decrease in sperm count and quality [31]. On the other hand, the gene *RNASE10* is highly expressed in the male reproductive system, especially in the epididymis. *RNASE10* plays an important role in the maturation process of spermatozoa and has an impact on male fertility [32]. The disruption of the *RNASE10* gene in mice results in defective sperm binding and altered motility [33]. Also, *RNASE10* has an effect on the development of the epididymis, and its expression in proximal epididymal cells leads to the selective inactivation of the androgen receptor (Ar) in the proximal principal cells of the epididymis, resulting in epithelial hypoplasia and atrophy [33]. Meanwhile, we found that the difference in the motility of the epididymal spermatozoa of Tibetan sheep at different altitudes was not significant and that the up-regulation of some genes in the TSH group could help spermatozoa maintain their motility. Among them, *ADCYAP1R1* can regulate sperm motility through the cAMP signaling pathway [34,35]. *CABP2* and *CALN1* can affect sperm motility by regulating calcium ion homeostasis and cellular signaling. *ATP6V1B1* has a crucial effect on sperm maturation and storage [36]. Relevant studies have shown that in epididymal tissues, clear cells in the epididymis maintain the expressions of a set of characteristic genes, including *FOXI1*, *KRT7*, and *ATP6V1B1* [37]. *ATP6V1B1* is specifically expressed in epididymal V-ATPase-rich clear cells, which are responsible for acidifying the lumen [38]. The knockdown or disruption of *ATP6V1B1* or other V-ATPase subunits impairs this acidification process, leading to sterility and sperm maturation defects [39]. We suggest that the more transparent cells of the epididymal epithelium are required to maintain the stability of the epididymal luminal pH [40] for sperm maturation [41] and survival in high-altitude environments. Further functional annotation analysis of the differential genes showed that most of the genes were enriched in the GO database for processes related to spermatogenesis and maturation, which is consistent with the findings of Li, S. et al. [42,43]. In addition, we found that differentially expressed genes were enriched in cilia- or flagellum-dependent cell motility and sperm motility, thus affecting sperm motility. We speculate that some biological processes and metabolic functions of the epididymis change at different altitudes and result in corresponding physiological and behavioral responses to adapt to the special environment, making the differences in sperm motility between different altitudes not significant. In addition, KEGG functional enrichment analysis of differential genes showed differential calcium signaling pathways, cAMP signaling pathways, and cytokine–cytokine receptor interaction-related pathways. Notably, environmental information processing is the most dominant level in the caudal part of the epididymis of Tibetan sheep at different altitudes. It has been shown that spermatozoa motility receives different pathway regulation in high-altitude hypoxic environments [25]. *GRIA1*, fully known as AMPA ionotropic glutamate receptor 1 [44], is one of the core subunits of the AMPA (a-amino-3-hydroxy-5-methyl1-4-isoxazolepropenoic acid) receptor. A single AMPA receptor subunit has either a long or short intracellular c-terminal structural domain that binds different interacting proteins and affects AMPA receptor biosynthesis, transport, cellular scaffolding, and signaling [45]. It regulates follicle-stimulating hormone (FSH) and luteinizing hormone (LH) secretion by controlling the release of gonadotropin-releasing hormone. The epididymis serves as a target organ for FSH and LH, and FSH regulates the morphology of epididymal epithelial cells and the production of epididymal steroids in vitro [25]. We found that the SOD content in the TSL group was significantly higher than that in the TSH group. Studies have shown that hypoxia can reduce SOD activity [46]. Furthermore, the activities of antioxidant enzymes in the seminal plasma of male donkeys were positively correlated with their sperm motility [47]. This is consistent with the results in this paper.

We collected non-targeted metabolomics data from two groups of Tibetan sheep grown at different altitudes with consistent feeding management. We performed KEGG pathway analysis on the differentially expressed metabolites. The enrichment of key molecules in these pathways suggests relatively high cAMP levels in low-altitude Tibetan sheep. In addition, we found that ATP and adenosine triggered the accumulation of proton pump ATPase (V-ATPase) in the apical membrane, leading to the acidification of the lumen, which is critical for sperm maturation and storage [48]. In addition, adenosine transmits energy in the form of adenosine triphosphate (ATP), adenosine diphosphate (ADP), or cyclic adenosine monophosphate (cAMP) to facilitate signal transduction. Moreover, prostaglandin I2 can induce cAMP accumulation and stimulate testis and epididymal sperm motility in the presence of ATP, cAMP, appropriate calcium levels, and pH [49]. Notably, the cAMP signaling pathway and the VEGF signaling pathway are key metabolic pathways in the adaptation of animals to high-altitude environments and play crucial roles in the caudal development of the epididymis and sperm maturation in Tibetan sheep.

The regulation of differentially expressed genes (DEGs) and differentially metabolites (DMs) in the epididymal tails of Tibetan sheep grown at different altitudes was investigated. The results show that DEGs involved in the regulation of the cAMP signaling pathway, such as *ADCYAP1R1*, *CHRM2*, *GRIA1*, and *GRIA3*, were also involved in neuroactive ligand–receptor interactions. The DMs identified in this study were prostaglandin I2 and L-lactate. Interestingly, prostaglandin I2 is involved in the VEGF signaling pathway and neuroactive ligand–receptor interactions. Prostaglandin I2 promotes angiogenesis by increasing the expression of vascular endothelial growth factor (VEGF) through the activation of peroxisomal enzymes [50]. At the same time, glycolysis, an essential energy supply pathway in animal cell metabolism [51], was analyzed jointly through the transcriptome and metabolome and involves the conversion of glucose, fructose, and other sugars to pyruvate via the corresponding transporter proteins in the cytoplasm, followed by a series of biochemical reactions. In this study, we found a significant correlation between the GRIA1 gene and D-2-phosphoglyceric acid. 1,3-Diphospho-D-glycerate is converted to D-2-phosphoglyceric acid by phosphoglycerate kinase (PGK) to release ATP. This represents the first substrate-level phosphorylation in the process of glycolysis that generates ATP. D-2-phosphoglyceric acid is a key glycolytic metabolite [52], and its higher concentration in the TSH group suggests that increased glycolytic activity in high-altitude Tibetan sheep improves sperm motility in low-oxygen environments at high altitudes. However, D-2-phosphoglyceric acid was positively correlated with sperm deformation rate. Only when its position is at a certain level can the sperm motility be maintained and the malformation rate be reduced.

## 5. Conclusions

Through transcriptomic and metabolomic analyses, we found that the epididymal tails of Tibetan sheep raised at different altitudes exhibited differential gene expressions, mainly involving processes related to reproduction, immunity, and membrane components. The differentially expressed genes were significantly enriched in key signaling pathways, such as the calcium signaling pathway, cAMP signaling pathway, and cytokine–cytokine receptor interactions, which are crucial for maintaining sperm survival rate and motility.

## Figures and Tables

**Table 1:** Comparison of hormone contents in Tibetan sheep at different altitudes.

Hormones	TSH	TSL	*p*-Value
FSH	3.33 ± 0.73	3.52 ± 0.43	*p* ≥ 0.05
T	3.44 ± 0.05	3.45 ± 0.19	*p* ≥ 0.05
SOD	1.7 ± 0.14	2.36 ± 0.27	*p* < 0.01

## Data Availability

The datasets presented in this study can bAe found in online repositories. The names of the repository/repositories and accession number(s) can be found at NCBI, PRJNA1043908.

## Supplementary Materials

The following supporting information can be downloaded at https://www.mdpi.com/article/10.3390/ani14213117/s1, Table S1: Primer sequences for qRT-PCR; Table S2: Statistical table for filtering RNA-Seq data.

## References

[1] Zhao P.F., Li S.B., He Z.H., Zhao F.F., Wang J.Q., Liu X., Li M.N., Hu J., Zhao Z.D., Luo Y.Z. (2022). Physiology and Proteomic Basis of Lung Adaptation to High-Altitude Hypoxia in Tibetan Sheep. Animals.

[2] Guo Y., Yang Y., Wang B., Liu C., Li M., Wang X. (2021). Morphological and scanning electron microscopic study of the gonadal arterioles in the Tibetan sheep. Anat. Histol. Embryol..

[3] Liu M., Yang Y., Li M., Cai Y., Zhao S., Wang X. (2022). Comparison study of morphology and vascular casts between plateau-type Tibetan sheep and low-altitude small-tailed Han sheep testes. Anat. Histol. Embryol..

[4] Chen H., Pu L., Tian C., Qi X., Song J., Liao Y., Mo B., Li T. (2024). Exploring the Molecular Characteristics and Role of PDGFB in Testis and Epididymis Development of Tibetan Sheep. Vet. Sci..

[5] Li T., Wang H., Luo R., Shi H., Su M., Wu Y., Li Q., Ma K., Zhang Y., Ma Y. (2023). Identification and Functional Assignment of Genes Implicated in Sperm Maturation of Tibetan Sheep. Animals.

[6] Liu J., Yao T., Weng X., Yao R., Li W., Xie L., Yue X., Li F. (2022). Antioxidant properties and transcriptome of cauda epididymis with different levels of fertility in Hu lambs. Theriogenology.

[7] Abdel-Maksoud F.M., Zayed A.E., Abdelhafez E.A., Hussein M.T. (2024). Seasonal variations of the epididymis in donkeys (*Equus asinus*) with special reference to blood epididymal barrier. Microsc. Res. Tech..

[8] Cheng P., Wei J., Liu B., Zhao Y., Ma B., Feng X., Xiong M., Zhao J., Shi C., Li Z. (2024). Metastasis-associated protein 1 participates in regulating luminal acidification of the epididymis via repressing estrogen receptor alpha transcription. Andrology.

[9] Lang X., Adjei M., Wang C., Chen X., Li C., Wang P., Pan M., Li K., Shahzad K., Zhao W. (2022). RNA-Seq reveals the functional specificity of epididymal caput, corpus, and cauda genes of cattleyak. Anim. Sci. J..

[10] Zhao W., Ahmed S., Liu J., Ahmed S., Quansah E., Solangi T.H., Wu Y., Yangliu Y., Wang H., Zhu J. (2021). Comparative iTRAQ proteomics identified proteins associated with sperm maturation between yak and cattleyak epididymis. BMC Vet. Res..

[11] Jin Y.Q., Fan Y.T., Sun H., Zhang Y., Wang H.R. (2021). Transcriptome Analysis Reveals Catabolite Control Protein A Regulatory Mechanisms Underlying Glucose-Excess or -Limited Conditions in a Ruminal Bacterium, *Streptococcus bovis*. Front. Microbiol..

[12] Zhang A.H., Sun H., Yan G.L., Wang P., Wang X.J. (2016). Mass spectrometry-based metabolomics: Applications to biomarker and metabolic pathway research. Biomed. Chromatogr..

[13] Gu L., Li S., Zhang R., Zhang Y., Wang X., Zhang K., Liu Z., Bi K., Chen X. (2015). Integrative investigation of *Semen Strychni* nephrotoxicity and the protective effect of *Radix Glycyrrhizae* by a UPLC-MS/MS method based cell metabolomics strategy in HEK 293t cell lysates. RSC Adv..

[14] Narud B., Khezri A., Kvitvang H.F., Klinkenberg G., Zeremichael T.T., Stenseth E.B., Myromslien F.D., Kommisrud E. (2018). Age effect on semen quality, metabolomics and fertility in young Norwegian Red bulls. Reprod. Domest. Anim..

[15] Park J., Lee C., Park S., Kim Y.S., Suh Y. (2016). DNA fingerprint, metabolomics and microscopy provide new evidence for the standardization of Armeniacae semen and Persicae semen. Planta Medica.

[16] Rosa R., Costa P.R., Pereira J., Nunes M.L. (2004). Biochemical dynamics of spermatogenesis and oogenesis in Eledone cirrhosa and Eledone moschata (Cephalopoda: Octopoda). Comp. Biochem. Physiol. Part B Biochem. Mol. Biol..

[17] Silvestroni L., Frajese G., Fabrizio M. (1976). Histones instead of protamines in terminal germ cells of infertile, oligospermic men. Fertil. Steril..

[18] Lin Y., Li J., Wang K., Fang Z., Che L., Xu S., Feng B., Zhuo Y., Li J., Wu D. (2022). Effects of dietary L-leucine supplementation on testicular development and semen quality in boars. Front. Vet. Sci..

[19] Partyka A., Rodak O., Bajzert J., Kochan J., Nizanski W. (2017). The Effect of L-Carnitine, Hypotaurine, and Taurine Supplementation on the Quality of Cryopreserved Chicken Semen. Biomed Res. Int..

[20] Kiyamu M., Bigham A., Parra E., Leon-Velarde F., Rivera-Chira M., Brutsaert T.D. (2012). Developmental and genetic components explain enhanced pulmonary volumes of female peruvian quechua. Am. J. Phys. Anthropol..

[21] Zhao P., Zhao F., Hu J., Wang J., Liu X., Zhao Z., Xi Q., Sun H., Li S., Luo Y. (2022). Physiology and Transcriptomics Analysis Reveal the Contribution of Lungs on High-Altitude Hypoxia Adaptation in Tibetan Sheep. Front. Physiol..

[22] Soliman M.M., Aldhahrani A., Althobaiti F., Ahmed M.M., Sayed S., Alotaibi S., Shukry M., El-Shehawi A.M. (2022). Characterization of the Impacts of Living at High Altitude in Taif: Oxidative Stress Biomarker Alterations and Immunohistochemical Changes. Curr. Issues Mol. Biol..

[23] Li W., Zeng W., Jin X., Xu H., Fang X., Ma Z., Cao G., Li R., Ma L. (2022). High-Altitude Stress Orchestrates mRNA Expression and Alternative Splicing of Ovarian Follicle Development Genes in Tibetan Sheep. Animals.

[24] Li X.-Y., Zhang M.-H., Chen Z.-W., Zhang B., Bai G., Wang H.-F. (2023). Male reproductive system and simulated high-altitude environment: Preliminary results in rats. Asian J. Androl..

[25] Verratti V., Di Giulio C., D’Angeli A., Tafuri A., Francavilla S., Pelliccione F. (2016). Sperm forward motility is negatively affected by short-term exposure to altitude hypoxia. Andrologia.

[26] Kastelic J.P., Wilde R.E., Bielli A., Genovese P., Rizzoto G., Thundathil J. (2019). Hyperthermia is more important than hypoxia as a cause of disrupted spermatogenesis and abnormal sperm. Theriogenology.

[27] Vargas A., Bustos-Obregón E., Hartley R. (2011). Effects of hypoxia on epididymal sperm parameters and protective role of ibuprofen and melatonin. Biol. Res..

[28] Zeng J., Gao W., Tang Y., Wang Y., Liu X., Yin J., Su X., Zhang M., Kang E., Tian Y. (2023). Hypoxia-sensitive cells trigger NK cell activation via the KLF4-ASH1L-ICAM-1 axis, contributing to impairment in the rat epididymis. Cell Rep..

[29] Nabi S., Askari M., Rezaei-Gazik M., Salehi N., Almadani N., Tahamtani Y., Totonchi M. (2022). A rare frameshift mutation in *SYCP1* is associated with human male infertility. Mol. Hum. Reprod..

[30] Schilit S.L., Menon S., Friedrich C., Kammin T., Wilch E., Hanscom C., Jiang S., Kliesch S., Talkowski M.E., Tüttelmann F. (2020). SYCP2 Translocation-Mediated Dysregulation and Frameshift Variants Cause Human Male Infertility. Am. J. Hum. Genet..

[31] Kolas N.K., Yuan L., Hoog C., Heng H.H., Marcon E., Moens P.B. (2004). Male mouse meiotic chromosome cores deficient in structural proteins SYCP3 and SYCP2 align by homology but fail to synapse and have possible impaired specificity of chromatin loop attachment. Cytogenet. Genome Res..

[32] Fujihara Y., Noda T., Kobayashi K., Oji A., Kobayashi S., Matsumura T., Larasati T., Oura S., Kojima-Kita K., Yu Z. (2019). Identification of multiple male reproductive tract-specific proteins that regulate sperm migration through the oviduct in mice. Proc. Natl. Acad. Sci. USA.

[33] Krutskikh A., De Gendt K., Sharp V., Verhoeven G., Poutanen M., Huhtaniemi I. (2011). Targeted Inactivation of the Androgen Receptor Gene in Murine Proximal Epididymis Causes Epithelial Hypotrophy and Obstructive Azoospermia. Endocrinology.

[34] Muslim A., Zulkarnain Z., Gholib G., Teuku Zahrial H., Sugito S. The Prominent Role of Pituitary Adenylate Cyclase-Activating Polypeptide in Spermatogenesis and Function of Spermatozoa: A Mini Review. Proceedings of the 2nd International Conference on Veterinary, Animal, and Environmental Sciences (ICVAES 2020).

[35] Winters S.J., Moore J.P. (2020). PACAP: A regulator of mammalian reproductive function. Mol. Cell. Endocrinol..

[36] Blomqvist S.R., Vidarsson H., Söder O., Enerbäck S. (2006). Epididymal expression of the forkhead transcription factor Foxi1 is required for male fertility. EMBO J..

[37] Pou Casellas C., Pleguezuelos-Manzano C., Rookmaaker M.B., Verhaar M.C., Clevers H. (2023). Transcriptomic profile comparison reveals conservation of ionocytes across multiple organs. Sci. Rep..

[38] Da Silva N., Pisitkun T., Belleannée C., Miller L.R., Nelson R., Knepper M.A., Brown D., Breton S. (2010). Proteomic analysis of V-ATPase-rich cells harvested from the kidney and epididymis by fluorescence-activated cell sorting. Am. J. Physiol. Cell Physiol..

[39] Roy J.W., Hill E., Ruan Y.C., Vedovelli L., Păunescu T.G., Brown D., Breton S. (2013). Circulating aldosterone induces the apical accumulation of the proton pumping V-ATPase and increases proton secretion in clear cells in the caput epididymis. Am. J. Physiol. Cell Physiol..

[40] Finberg K.E., Wagner C.A., Bailey M.A., Paunescu T.G., Breton S., Brown D., Giebisch G., Geibel J.P., Lifton R.P. (2005). The B1-subunit of the H(+) ATPase is required for maximal urinary acidification. Proc. Natl. Acad. Sci. USA.

[41] Nishie M., Suzuki E., Hattori M., Kawaguch K., Kataoka T.R., Hirata M., Pu F., Kotake T., Tsuda M., Yamaguchi A. (2021). Downregulated *ATP6V1B1* expression acidifies the intracellular environment of cancer cells leading to resistance to antibody-dependent cellular cytotoxicity. Cancer Immunol. Immunother. CII.

[42] Li S., Yang Q.-E. (2022). Hypobaric hypoxia exposure alters transcriptome in mouse testis and impairs spermatogenesis in offspring. Gene.

[43] Li Y., Li R.-H., Ran M.-X., Zhang Y., Liang K., Ren Y.-N., He W.-C., Zhang M., Zhou G.-B., Qazi I.H. (2018). High throughput small RNA and transcriptome sequencing reveal capacitation-related microRNAs and mRNA in boar sperm. BMC Genom..

[44] Maksimovic M., Vekovischeva O.Y., Aitta-aho T., Korpi E.R. (2014). Chronic Treatment with Mood-Stabilizers Attenuates Abnormal Hyperlocomotion of GluA1-Subunit Deficient Mice. PLoS ONE.

[45] Groc L., Choquet D. (2006). AMPA and NMDA glutamate receptor trafficking: Multiple roads for reaching and leaving the synapse. Cell Tissue Res..

[46] Simon L.M., Liu J., Theodore J., Robin E.D. (1977). Effect of hyperoxia, hypoxia, and maturation on superoxide dismutase activity in isolated alveolar macrophages. Am. Rev. Respir. Dis..

[47] Papas M., Arroyo L., Bassols A., Catalán J., Bonilla-Correal S., Gacem S., Yeste M., Miró J. (2019). Activities of antioxidant seminal plasma enzymes (SOD, CAT, GPX and GSR) are higher in jackasses than in stallions and are correlated with sperm motility in jackasses. Theriogenology.

[48] Futai M., Oka T., Sun-Wada G., Moriyama Y., Kanazawa H., Wada Y. (2000). Luminal acidification of diverse organelles by V-ATPase in animal cells. J. Exp. Biol..

[49] Bennegard B., Hahlin M., Hamberger L. (1990). Luteotropic effects of prostaglandins I2 and D2 on isolated human corpora luteum. Fertil. Steril..

[50] Buchanan F.G., Chang W., Sheng H., Shao J., Morrow J.D., DuBois R.N. (2004). Up-regulation of the enzymes involved in prostacyclin synthesis via Ras induces vascular endothelial growth factor. Gastroenterology.

[51] Wen Y., Hu J., Wang J., Liu X., Li S., Luo Y. (2021). Effect of glycolysis and heat shock proteins on hypoxia adaptation of Tibetan sheep at different altitude. Gene.

[52] Marshall C.J., Garrett K., Van Vliet S., Beck M.R., Gregorini P. (2022). Dietary and Animal Strategies to Reduce the Environmental Impact of Pastoral Dairy Systems Result in Altered Nutraceutical Profiles in Milk. Animals.

