# Supplementary material for: Transcriptome and Metabolome Analyses Reveal High-Altitude Adaptation Mechanism of Epididymis Sperm Maturation in Tibetan Sheep
Source: Animals (Basel). 2024 Oct 29;14(21):3117. doi: 10.3390/ani14213117 (PMC11544902; doi:10.3390/ani14213117)
Supplement: Supplementary file 1 [file animals-14-03117-s001.zip › animals-3201534-supplementary.pdf]

Table S1: Primer sequences for qRT-PCR

Gene	Primer sequences
LOC121819736	F: AGGATTTTTCTCTGTTCGTGAGT<br>R: TATCGGAGGAAAGCAGAGCAA
LOC10111092	F: CTGGCTGCTACAGGGTTCTG<br>R: AGTGCGTCTTGAACACGTTG
SL18A2	F: CAGCGCATGGTCACTAACTC<br>R: ACATGGGAATTGGGTAGCCA
NOTUM	F: AATGCCAACATGGTCTTC<br>R: CGTACTCGTTCTTCTCAG
PI3	F: GCAGAGGCAGCTATCATA<br>R: ACAGGATATTGACCGTTGA
MYBPH	F: GATTCAGGCTGCTATGAG<br>R: TCAATCACCAGGATGTTG
CSMD1	F: TAGTGGTCTTGAGTGGAA<br>R: GCTGAATAACGAATCTTGAAT
DPYS	F: GATGCTGACATTGTGATT<br>R: CGAAGATGTTGAAGTTGA
GRIA1	F: GAAGCGGGATCTACGAAGGA<br>R: GGATCATCCCCTCCTCTGTG
β-actin	F: TGATGATCGCAGAAAGAACCC<br>R: CTCGCTTTGAAGGTTTCCAGT

Table S2: Statistical table for filtering RNA-Seq data

Sample	Raw_Read_Number	Raw_Bases	Raw_Q30_number	Raw_N_rate	Raw_Q20_rate	Raw_Q30_rate
ZYH1	49301270	7444491770	7094708645	0.003477	98.43	95.3
ZYH2	46731186	7056409086	6653794331	0.003509	98.03	94.29
ZYH3	52060208	7861091408	7472252598	0.003482	98.33	95.05
ZYH4	50132276	7569973676	7162987750	0.00342	98.16	94.62
ZYH5	52542968	7933988168	7493927387	0.003365	98.09	94.45
ZYH6	55154280	8328296280	7863332148	0.003365	98.08	94.42
ZYM1	57748878	8720080578	8227620292	0.003385	98.05	94.35
ZYM2	50031806	7554802706	7153826116	0.00334	98.18	94.69
ZYM3	51614622	7793807922	7400277542	0.003333	98.28	94.95
ZYM4	41710986	6298358886	5954506042	0.003376	98.12	94.54
ZYM5	49866786	7529884686	7099789441	0.003401	98.02	94.29
ZYM6	54614526	8246793426	7782468072	0.003369	98.07	94.37
